# Comparison of three different methods for risk adjustment in neonatal medicine

**DOI:** 10.1186/s12887-017-0861-5

**Published:** 2017-04-17

**Authors:** Mark Adams, Julia Braun, Hans Ulrich Bucher, Milo Alan Puhan, Dirk Bassler, Viktor Von Wyl, The Swiss Neonatal Network, Philip Meyer, Claudia Anderegg, Sven Schulzke, Mathias Nelle, Bernhard Wagner, Thomas Riedel, Gregor Kaczala, Riccardo E. Pfister, Jean-Francois Tolsa, Matthias Roth-Kleiner, Martin Stocker, Bernhard Laubscher, Andreas Malzacher, John P. Micallef, Lukas Hegi, Dirk Bassler, Romaine Arlettaz, Vera Bernet

**Affiliations:** 1 Division of Neonatology, University Hospital Zurich and University of Zurich, Wagistrasse 14, 8952 Schlieren, Switzerland; 2 Epidemiology, Biostatistics and Prevention Institute, University of Zurich, Zurich, Switzerland

**Keywords:** Risk adjustment, Quality improvement, Neonatology, Effectiveness, Indirect standardization, Logistic regression, Multilevel, Mean brier score, and ROC area under curve

## Abstract

**Background:**

Quality improvement in health care requires identification of areas in need of improvement by comparing processes and patient outcomes within and between health care providers. It is critical to adjust for different case-mix and outcome risks of patient populations but it is currently unclear which approach has higher validity and how limitations need to be dealt with. Our aim was to compare 3 approaches towards risk adjustment for 7 different major quality indicators in neonatal intensive care (21 models).

**Methods:**

We compared an indirect standardization, logistic regression and multilevel approach. Parameters for risk adjustment were chosen according to literature and the condition that they may not depend on processes performed by treating clinics. Predictive validity was tested using the mean Brier Score and by comparing area under curve (AUC) using high quality population based data separated into training and validation sets. Changes in attributional validity were analysed by comparing the effect of the models on the observed-to-expected ratios of the clinics in standardized mortality/morbidity ratio charts.

**Results:**

Risk adjustment based on indirect standardization revealed inferior c-statistics but superior Brier scores for 3 of 7 outcomes. Logistic regression and multilevel modelling were equivalent to one another. C-statistics revealed that predictive validity was high for 8 and acceptable for 11 of the 21 models. Yet, the effect of all forms of risk adjustment on any clinic’s comparison with the standard was small, even though there was clear risk heterogeneity between clinics.

**Conclusions:**

All three approaches to risk adjustment revealed comparable results. The limited effect of risk adjustment on clinic comparisons indicates a small case-mix influence on observed outcomes, but also a limited ability to isolate quality improvement potential based on risk-adjustment models. Rather than relying on methodological approaches, we instead recommend that clinics build small collaboratives and compare their indicators both in risk-adjusted and unadjusted form together. This allows qualitatively investigating and discussing the residual risk-differences within networks. The predictive validity should be quantified and reported and stratification into risk groups should be more widely used to correct for confounding.

**Electronic supplementary material:**

The online version of this article (doi:10.1186/s12887-017-0861-5) contains supplementary material, which is available to authorized users.

## Background

Risk adjustment is ubiquitously used to compare health care providers for the identification of quality improvement potential. Its effectivity is usually accepted as given. Literature reports an abundance of variations in vital patient outcome measures between clinics, regions and even networks, as shown, for instance, in neonatology [1–3]. Could these clinics, regions or networks all achieve the same potentially best standard, the resulting effect would dwarf many successful introductions of new drugs or methods. Over the last 20 years, several neonatal networks have shown beneficial developments for patients that were associated with quality improvement efforts [4–7].

A number of publications have addressed the need for risk adjustment in quality improvement [8–10]. Several of the introduced models rely on processes or diagnostics performed by the site under observation, thereby inadvertently adjusting for factors that may well be in need of improvement [11].

The use of risk-adjustment thereby assumes that the discrepancy between predicted and observed outcome is at least partially attributable to the quality of care provided. If the observed outcome exceeds the predicted outcome, then this discrepancy is assumed to be due to poor care. To which degree this assumption is valid is an important but often neglected aspect of the validation of risk-adjustment methods [12, 13]. Literature therefore recommends observing predictive and attributional validity to assess the validity of the risk adjustment approaches. The predictive validity is the extent to which the method accurately predicts the probability of outcome whereas the attributional validity allows attributing differences in outcome to the quality of care [12].

As a national neonatal network, our aim was to setup a reliable, clinic-independent, achievable quality improvement concept for several vital outcome measures and test its validity. For this, we compared 3 approaches for adjusting our 7 most important outcome measures for very preterm born children and analysed their effect on the differences that the clinics show for these outcomes.

## Methods

### Study population

The study includes data on all children born alive between 22 0/7 and 31 6/7 weeks gestational age during the years 2006 to 2014 that were prospectively collected in our national, population based registry (SwissNeoNet). Data are collected electronically from birth until death or first discharge home by all nine Level III and three of six Level IIB neonatal units using immediate plausibility and completeness checks and subsequent repeated challenge until corrected. All items are defined in a publicly available manual [14]. They cover typical aspects of perinatal care, demographics, common diagnoses and treatments, growth and hospitalization duration. In this study we include all infants born between 2006 and 2014. Population coverage was assessed by comparison with the birth registry of the Swiss Federal Statistical Office yielding 94% completeness when including births of non-resident mothers living outside Switzerland.

### Outcome definitions

We selected outcomes that were strictly defined and whose incidence was alterable by variations in process or structure [9, 15]. Overall mortality was calculated for all infants born alive. The following proportions of new-borns with the respective outcome (i.e. cumulative incidence proportion over a 9-year time span) were based on infants admitted to a NICU: in-hospital mortality; late onset sepsis with clear clinical evidence of infection as well as at least one microbiologically relevant positive blood culture occurring after day 3 of life (with day of birth as day 1); necrotizing enterocolitis (NEC) was defined as clinical signs (abdominal distension, bilious aspirates and/or bloody stools) confirmed by radiographically visible intramural gas or at laparotomy (Bell stages 2 and 3); [16] severe intraventricular haemorrhage (sIVH) was based on the most severe ultrasound result during hospital stay using stages 3 to 4 of the classifications defined by Papile et al. [17]. The remaining outcome incidence proportions were based on infants discharged home alive: severe retinopathy of prematurity (sROP) using the international classification published by the committee for the classification of ROP grades 3–4; [18] and bronchopulmonary dysplasia (BPD) defined as an oxygen requirement at 36 weeks gestational age according to the NICHD consensus conference paper [19].

### Risk adjustment parameter definitions (risk adjustors)

We selected risk adjustors that were first and foremost predictive, i.e. known to vary with changes in severity in outcomes. Beyond this, they were easily available to restrict bias due to missing data, measurable, frequent, reliable, and accurately recorded with limited interpretation margins to minimize definition bias [9]. Another selection criterion was that they were consistent with literature, i.e. that their validity has already been documented for the listed outcomes [10, 11, 20]. Our risk adjusters also hold the ability to be updated periodically, based on an ongoing research commitment and investment [8].

The following risk adjusters were included in the model: Gestational age (GA), GA squared (GA^2^), birthweight z-score (BW z-score), male sex, multiples, Apgar at 1 min, major malformation, outborn and parent socio economic status (SES). GA was calculated based on ultrasound examinations during the first trimester of pregnancy and defined as postmenstrual age in weeks and days. Major congenital malformation was defined as any type of malformation severely impacting prognosis (e.g. complex congenital heart disease, malformation syndromes). SES was estimated by a validated 12-point socioeconomic score based on maternal education and current paternal occupation whereas the value 2 designates the combination of highest education and occupation versus the value 12 which represents no education and no occupation [21]. Outborn designates an infant transferred to any one of the 12 participating units after birth.

### Statistical methods

Based on the risk-adjustment methodology published in literature, three approaches were compared: indirect standardization based on the units’ individual distribution of children into gestational age weeks, the most commonly used multivariable logistic regression to account for multiple confounding variables, and multivariable multilevel modelling [5, 22, 23]. The latter was added in case clustering of data into neonatal units should be of importance. A previous publication revealed that the survival of Swiss infants with very low gestational age without severe neonatal morbidity was strongly influenced by the medical centre that treated them [24]. Data of infants born from 2006 to 2012 were used for model building (training set) whereas data from 2013 to 2014 were used for goodness-of-fit analysis (validation set). This split was performed to avoid overfitting, to be able to use the best acquired model on our own data and to accurately describe what effect each model has on the observed-to-expected ratios of the clinics.

In order to avoid collinearity between GA and birthweight, birthweight entered the model as z-scores relative to GA based on the growth curves published by Voigt et al. [25]. To better model the non-linear dependency of most neonatal outcomes on gestational age, its quadratic value (GA^2^) was included into the latter 2 models. As several of the examined outcomes are known to have improved over the years, [26] year was added as a covariate as a continuous variable assigning the value 0 to the last year (2012) and subtracting 1 for each previous year (value range [−6, 0]); this covariate adjusts the remaining predictors for a linear effect of time. It was omitted in validation.

As one of the predictors, i.e. SES, yielded 20% missing values in the entire dataset, equally distributed over the years but with higher concentration on datasets of infants that died in the delivery room, we first calculated the models using fivefold imputation with chained equations [27]. The results were compared to those resulting from models without imputation and without SES as predictor. Models with imputation and SES were used only if SES yielded a significant adjusted odds ratio (i.e. 95% confidence interval outside 1). All other predictors yielded less than 2% missing data and were therefore not treated separately. No other model selection process was undertaken.

In the multilevel models, centre was included as a random intercept to adjust for centre differences while providing parameter estimates to permit centre-free predictions [20]. As the compared units were all of the same standard (swiss perinatal units are legally required to fulfil the same structural qualifications on staffing and equipment), we did not add any cluster level covariates. All statistical analyses were performed using R [28]. *P*-values below 0.05 were considered significant.

### Validity assessments

To compare predictive validity of the different adjustment approaches, we compared the mean Brier score and the area under receiver operating characteristics curve (AUC). The Brier score measures the accuracy of probabilistic predictions as the mean squared difference between the predicted probability assigned to the possible outcome and the actual outcome. The closer the score is to 0, the better the sharpness and calibration of the prediction. A poor Brier score of 0.25 is reached by assigning each patient with a constant probability of 0.5 for the outcome [29]. AUC values between 0.7–0.8 were considered to represent moderate predictive validity whereas and >0.8 to represent high predictive validity. As no goodness-of-fit can be calculated for indirect standardization, comparison was approximated using a logistic regression model based on gestational weeks alone. The difference in prediction between both was less than 0.5% (Additional file 1: Table S1).

As we were not able to measure attributional validity, we instead measured changes to attributional validity by assessing what the effect of a specific risk adjustment model was on the differences seen in the risk-adjusted outcome measures. Standardized mortality / morbidity ratios were calculated as observed over expected ratios for each clinic. Expected outcome per clinic was calculated as sum of standardized outcome ratio per gestational age week in the case of indirect standardization and per each clinic’s sum of individual patient probabilities (0 < *p* < 1) to acquire a specific outcome in the case of logistic regression or multilevel modelling, respectively. Individual patient probability was calculated as P(Y = 1| X_i_=x_i_) = exp(β_0_+β_1_X_1_+ …  + β_n_X_n_)/1 + exp(β_0_+β_1_X_1_+ …  + β_n_X_n_).

## Results

Table 1 lists the 6 outcome variables and the proportion of new-borns presenting the respective outcome (i.e. cumulative incidence proportion) for which risk adjustment was performed. It also reveals the available data for prediction modelling and sensitivity analysis and the number of valid responses per outcome. Proportions are relative to the number of valid responses. There were sufficient data available for model building and testing. Other than severe ROP, for which 11% of data were estimated using data imputation, the outcomes had a maximum of ten missing valid responses.

**Table 1:** Outcomes observed, collective used, study population size (N), valid responses per outcome (valid N) and cumulative incidence proportions (%) for prediction modelling and sensitivity analysis

Variable	Collective	2006–2012			2013–2014		
		Training set			Validation set		
		N^a^	valid	%	N	valid	%
Mortality	All Live-born	5212	5212	14.6	1572	1572	14.0
In hospital mortality	Admitted to ward	4876	4874	8.8	1465	1465	7.7
Late onset sepsis			4874	9.5		1465	8.1
NEC^b^			4874	2.4		1465	2.4
sIVH^c^			4865	6.5		1459	5.7
BPD^d^	Discharged home alive	4449	4439	9.3	1352	1349	11.2
sROP^e^			3990	1.9		1200	1.6

^a^ *N* Sample size for analysis

^b^ *NEC* Necrotizing enterocolitis

^c^ *sIVH* intra−/periventricular haemorrhage grade 3–4

^d^ *BPD* bronchopulmonary dysplasia

^e^ *sROP* retinopathy of prematurity grade 3 and above

Table 2 compares the predictive validity of the three approaches for risk adjustment: indirect standardization based on GA stratification, logistic regression and multilevel modelling. Model parameters were calculated using the training set while prediction was tested using the validation set. For mortality, in hospital mortality and BPD, the indirect standardization had an inferior AUC but a superior Brier score. All other outcomes revealed close to identical Brier scores and a very similar AUC, whereas the values for logistic regression and multilevel modelling overall were almost identical. The variances of the random effect intercepts were all below 1.5 (not shown).

**Table 2:** Predictive validity for indirect standardization, logistic regression and multilevel random intercept approach respectively using the mean Brier score and AUC for assessing the predictive abilities of the respective model using the validation set

	Indirect Standardization		Logistic regression		Multilevel	
Variable	Brier Score	AUC	Brier Score	AUC	Brier Score	AUC
Mortality	0.070	0.896	0.113	0.935	0.109	0.936
In hosp. mortality	0.060	0.831	0.095	0.892	0.093	0.893
Late onset sepsis	0.068	0.788	0.067	0.804	0.067	0.803
NEC^a^	0.023	0.716	0.024	0.694	0.024	0.694
sIVH^b^	0.050	0.765	0.049	0.786	0.049	0.786
BPD^c^	0.084	0.807	0.195	0.843	0.195	0.843
sROP^d^	0.015	0.765	0.016	0.773	0.016	0.773

^a^ *NEC* Necrotizing enterocolitis. Values based on imputed data

^b^ *sIVH* intra−/periventricular haemorrhage grade 3–4

^c^ *BPD* bronchopulmonary dysplasia

^d^ *sROP* retinopathy of prematurity grade 3 and above. Values based on imputed data

The 3 risk adjustment approaches over 7 outcomes yielded 21 risk adjustment models. Nineteen of these models reached a Brier Score below 0.1. Equally 19 reached at least an acceptable predictive validity (AUC > 0.7) of which 11 even reached a high predictive validity (AUC > 0.8) (Table 2).

**Fig. 1:**
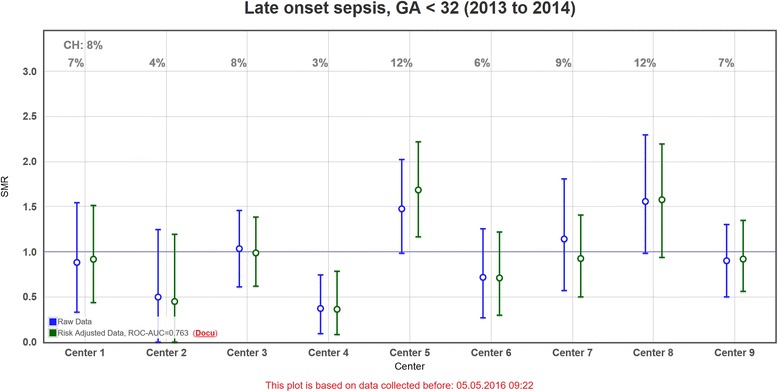
Standardized morbidity ratio chart displaying late onset sepsis prediction for 9 Swiss NICUs with 95% confidence intervals (screenshot from SwissNeoNet member platform). *Blue*: crude observed over expected data, *green*: risk adjusted relation of observed to expected deaths in hospital (including delivery room). Expected deaths were calculated as sum of each child’s probability to die depending upon the predictor values listed in Table 4

Next we looked at the effect each risk adjustment approach had on the distribution of the individual clinic’s outcomes in the standardized mortality / morbidity ratio (SMR) charts in order to ascertain if the differences in case-mix were effectively adjusted for and the remaining differences can be attributed to differences in care. SMR charts are ubiquitously used to analyse a clinic’s performance by comparing the risk adjusted ratio of observed to expected mortality / morbidity cases to a standard population as shown in Fig. 1. For this we first determined for each model the number of clinics in the validation set whose observed to expected ratio were above 1, i.e. worse than expected, the value that usually interests the clinicians most (Table 3a). Mortality, in-hospital mortality and BPD listed the same amount of SMR values above 1. The values for the other outcomes remained largely untouched. To determine if these values above 1 represent the same clinics, we analysed all of the 252 possible SMR changes generated out of the total of 7 (outcomes) × 9 (clinics) × 4 (approaches) (not shown). Of 252 possible changes of orientation between all approaches, a total of 14 changes were observed, 7 of which were between raw values and all other risk adjustment models, and 7 between indirect standardization and unadjusted as well as adjusted models. These changes of orientation concern clinics with observed to expected ratios close to one. There was no other fluctuation of clinics from above to below one due to risk adjustment. Our second analysis concerned the cumulative absolute distance of the observed to expected ratio from 1 for each approach in order to determine if the approaches lead to larger or smaller overall differences between clinics (Table 3b). Absolute here means that the distances of all clinics above and below one are summarized. The higher the value, the more the clinics seem to differ from each other. Again, the differences between the approaches per outcome are marginal but somewhat higher when using a logistic regression or multilevel approach. In order to exclude that the lack of effect of risk adjustment is based on a lack of difference between the clinics’ risk potential, we calculated the expected mortality for the clinic with the lowest risk in the validation set as 11.2% versus 15.9% for the clinic with the highest risk according to the logistic regression model (AUC 0.935). Thus, the risk heterogeneity between the two clinics lies at 40%.

**Table 3:** Effect of risk-adjustment approach on standardized mortality/morbidity ratio chart. a) Number of clinics with observed-expected ratio > 1. b) Cumulative absolute distances of observed-expected ratio of all clinics to the standard value 1

	Raw values	Indirect standardization	Logistic regression	Multilevel
Outcome	a) SMR values above 1			
Mortality	3	3	3	3
In hosp. Mortality	3	3	3	3
Late onset sepsis	4	2	2	2
NEC^a^	3	4	3	3
sIVH^b^	5	5	6	6
BPD^c^	4	4	4	4
sROP^d^	2	2	1	2
	b) Cumulative absolute distances to 1			
Mortality	2.8	2.7	3.0	3.0
In hosp. Mortality	3.0	3.2	3.1	3.0
Late onset sepsis	2.7	3.0	3.0	3.1
NEC	3.4	3.4	3.6	3.6
sIVH	2.2	2.3	2.4	2.4
BPD	4.1	3.2	3.8	3.8
sROP	5.7	5.4	5.4	5.5
Total	23.9	23.2	24.3	24.4

^a^ *NEC* Necrotizing enterocolitis

^b^ *sIVH* intra−/periventricular haemorrhage grade 3–4

^c^ *BPD* bronchopulmonary dysplasia

^d^ *sROP* retinopathy of prematurity grade 3 and above

Table 4 summarizes the adjusted odds ratios of the risk adjustors per approach (indirect standardization, logistic regression and multilevel). Our indirect standardization approach relies on gestational age stratification alone which explains the difference between the odds ratios per outcome in comparison with the other approaches. The risk adjustor’s odds ratios for the logistic regression and the multilevel approach are however largely congruent with only marginal differences, explaining the close comparability in the analyses above. The cumulatively strongest effect, i.e. the largest difference in relation to 1, is attributable to gestational age. A relatively high impact on some of the models is observable in major malformation. SES score only had an effect for the outcome NEC [OR 1.13, 95% CI 1.04–1.22] (note that the highest SES score (12) stands for lowest socio economic *status* whereas the lowest score (2) stands for the highest SES collected).

**Table 4:** Odds ratios (OR) with standard errors (SE) of the risk adjustment parameters for each outcome and each risk adjustment (RA) approach

Outcome	RA approach	GA^a^		GA^2^		BW^b^ z-score		Male sex		Multiples		Apgar1		Major malformation		outborn		SES^c^	
		OR	SE	OR	SE	OR	SE	OR	SE	OR	SE	OR	SE	OR	SE	OR	SE	OR	SE
Mortality	Indirect standardization	0.52	0.02	-	-	-	-	-	-	-	-	-	-	-	-	-	-	-	-
	Logistic regression	0.20	0.12	1.10	0.01	0.88	0.06	1.07	0.11	1.03	0.12	0.74	0.02	7.88	0.19	0.76	0.27	-	-
	Multilevel	0.19	0.12	1.10	0.01	0.84	0.06	1.09	0.11	1.03	0.12	0.74	0.02	8.62	0.20	0.74	0.28	-	-
In hosp. Mortality	Indirect standardization	0.57	0.03	-	-	-	-	-	-	-	-	-	-	-	-	-	-	-	-
	Logistic regression	0.31	0.15	1.06	0.01	0.81	0.06	1.13	0.11	1.08	0.13	0.79	0.02	4.65	0.24	0.94	0.27	-	-
	Multilevel	0.28	0.15	1.07	0.01	0.79	0.06	1.16	0.12	1.06	0.13	0.80	0.02	5.34	0.24	0.89	0.28	-	-
Late onset sepsis	Indirect standardization	0.68	0.02	-	-	-	-	-	-	-	-	-	-	-	-	-	-	-	-
	Logistic regression	1.11	0.14	0.96	0.01	0.71	0.06	0.95	0.10	0.83	0.12	1.03	0.02	1.32	0.28	1.08	0.24	-	-
	Multilevel	1.02	0.15	0.96	0.01	0.68	0.06	0.96	0.10	0.79	0.12	1.03	0.02	1.48	0.29	0.98	0.24	-	-
NEC^d^	Indirect standardization	0.81	1.04	-	-	-	-	-	-	-	-	-	-	-	-	-	-	-	-
	Logistic regression	1.12	1.30	0.98	1.02	0.78	1.11	1.12	1.21	1.13	1.23	0.91	1.04	1.00	1.69	1.30	1.46	1.11	1.04
	Multilevel	1.09	1.30	0.98	1.02	0.78	1.11	1.13	1.21	1.11	1.23	0.91	1.04	1.01	1.69	1.32	1.46	1.11	1.04
sIVH^e^	Indirect standardization	0.65	0.03	-	-	-	-	-	-	-	-	-	-	-	-	-	-	-	-
	Logistic regression	0.64	0.16	1.01	0.01	1.30	0.07	1.28	0.12	1.18	0.13	0.85	0.03	0.69	0.41	1.37	0.25	-	-
	Multilevel	0.63	0.16	1.01	0.01	1.31	0.07	1.27	0.12	1.19	0.13	0.85	0.03	0.69	0.41	1.33	0.25	-	-
BPD^f^	Indirect standardization	0.62	0.03	-	-	-	-	-	-	-	-	-	-	-	-	-	-	-	-
	Logistic regression	0.79	0.17	0.98	0.01	0.53	0.07	1.60	0.12	1.03	0.13	0.94	0.02	3.91	0.27	1.18	0.27	-	-
	Multilevel	0.73	0.17	0.99	0.01	0.52	0.07	1.60	0.12	1.01	0.13	0.94	0.02	4.40	0.27	1.11	0.27	-	-
sROP^g^	Indirect standardization	0.47	1.07	-	-	-	-	-	-	-	-	-	-	-	-	-	-	-	-
	Logistic regression	0.45	1.42	1.01	1.03	0.75	1.16	1.23	1.28	1.26	1.31	0.93	1.05	1.06	2.17	1.51	1.73	0.98	1.05
	Multilevel	0.35	1.44	1.03	1.03	0.75	1.16	1.31	1.28	1.06	1.31	0.94	1.05	0.97	2.19	1.56	1.76	0.98	1.05

^a^ *GA* gestational age

^b^ *BW* birthweight

^c^ *SES* socio-economic status

^d^ *NEC* Necrotizing enterocolitis

^e^ *sIVH* intra−/periventricular hemorrhage grade 3–4

^f^ *BPD* bronchopulmonary dysplasia

^g^ *sROP* retinopathy of prematurity grade 3 and above

## Discussion

In our study we analyse prospectively collected population based data with high population coverage. The aim was to develop and compare risk adjustment models for quality improvement purposes within a network of 12 Swiss neonatal units. The selected outcomes for risk adjustment represent some of the most important outcome measures in the field of neonatology [30]. We selected the two well-known and most often implemented approaches for risk adjustment, indirect standardization and logistic regression modelling and added a multilevel approach due to the nested data structure in which patients were associated with a particular clinic and its ability to provide clinic-independent predictions. Both prediction and outcome parameters fulfil multiple requirements for risk adjustment. Over half of the 21 risk adjustment models (three risk adjustment approaches for 7 outcomes each) yielded high predictive validity in the validation set with a majority of the remaining models being at least moderately predictive. In summary, the three risk adjustment approaches are similar to each other and lead to results that differ marginally when compared with the unadjusted raw outcome comparisons.

The simplest approach, indirect standardization based on GA, performed surprisingly well in comparison and may be more useful for quality assessments than previously thought. This may be due to the high impact of gestational age on outcome in very preterm infants and may not be generalizable into other areas of medicine. Nevertheless, reduction to one reliably available risk parameter may have removed bias that lead to an inferior accordance between predicted and true values in the multivariable approach. New medical registries without available previous data could profit from the observation that a non-model based approach achieves comparable if not better calibrated results to model based approaches. The lack of differences between the logistic regression and the multilevel approach and the small variances of the random effect intercepts of the multilevel model revealed that the effect of a nested data structure is lower than expected for a neonatal setting [20].

In summary, the three approaches reveal very little difference in predictive or attributional validity between each other. Nevertheless, their ability to isolate quality improvement potentials of the units is limited. This was unexpected because the predictive validities for the carefully selected models were high. Also, large differences between Swiss clinics are documented in literature [24] and by a heterogeneity in risk potential between the clinics of up to 40%. Nevertheless, neither of the risk adjustment models had a discernible effect on the observed to expected ratios of the seven outcome parameters, neither in their absolute orientation (above or below 1) or in their distance to 1 (1 meaning that observed and expected are equal). The differences between units that remains after risk adjustment can therefore not be attributed to the quality of care alone. In neonatology, for instance, there are factors that contribute to the differences between clinics other than risk or quality of care. Even though there are national guidelines toward treatment at the limit of viability, [31] the Swiss clinics are known to adopt different approaches towards the care of infants born below 25 weeks GA. Clinics with a more active approach towards resuscitation in the delivery room will have a lower mortality but a higher risk for morbidities as the decision about whether to provide active treatment at birth is a critical predictor of subsequent outcomes in itself [32]. This can partially be adjusted for by stratified analysis of outcome of infants receiving active treatment, in our case “admitted to ward”. But a more active approach which yields a higher risk may still lead to higher survival without impairment as recently shown by Rysavy et al. [33].

In 1997, Iezzoni reported on a series of studies comparing the effect of different severity of illness scores on risk adjustment and concluded that although hospitals vary in their unadjusted death rates, severity failed to explain these differences fully leaving the central question unresolved: “does severity adjustment isolate that residual quantity, namely quality of care differences across hospitals?” [34] Also using a severity score, Thomas et al. found that hospital mortality performance was significantly related to quality of care for only 3 of 10 conditions evaluated [35]. The primary finding of a recent Monte Carlo simulation on the relationship between the predictive validity of the risk-adjustment model and the accuracy of hospital quality reports found that the relationship was, at best, modest [36]. The same researchers maintain that even if perfect risk-adjustment was possible, random error will result in some hospitals being misclassified [37]. The main rationale behind their claims is that the often used AUC is a measure of discrimination: the degree to which the model can discriminate between those patients with the outcome of interest and those without the outcome of interest. As a consequence it will be higher in a setting in which there is greater heterogeneity in risk [36]. Thus, models with low risk heterogeneity will be read as having low predictive validity regardless of their ability to adjust for risk.

Nevertheless, most published approaches to risk adjustment still rely exclusively on factors known for their high predictive validity such as severity of illness scores like SNAP or CRIB in neonatology [11]. These examples reflect NICU practices that were common at the time of their development (1993), require up to 28 physiological data characteristics from up to the first 24 h of the new-born’s lives, a period during which they are in the care of the clinics under observation for quality of performance, and require blood whereas differences in the timing of blood taking may affect risk adjustment [8]. Another hindering issue is the difficulty to achieve data completeness for all infants.

The SwissNeoNet selection of risk adjusters is therefore geared towards predictive validity, availability, and measurability and is the result of a consensus process among network members, by agreeing which factors the clinics should not be held responsible for in quality improvement and which factors should not be corrected for as they are under the control of the clinics. For the latter reason, we did not include caesarean section or antenatal steroids because we did not want to attribute a higher risk to clinics with lower rates in either of them. Particularly in the case of antenatal steroids, the units would have lost an opportunity to isolate a potential for improvement. Because the effect of risk adjustment is limited, members of the SwissNeoNet compare their outcomes in standardized mortality / morbidity ratio charts for stratified collectives and over pooled years. Stratifying can involve limiting the evaluation to a specific patient group such as from overall mortality to in-hospital-mortality, or separate analysis for infants born in different GA or birthweight groups. The crude observed-expected ratio (together with its 95% confidence interval) is displayed alongside its risk adjusted correspondent (Fig. 1). We also add the AUC value and link to documentation for the interpretation of the diagrams.

The strengths of our study lie in the quality and completeness of our population based data, in the data being covered for all live-born infants until death or primary discharge home, and in separating the data into training and validation sets. Switzerland provides wide regional diversity expressed by 26 cantons with 26 health care authorities, 4 languages, and 40% inhabitants with migration background. This diversity is also reflected in the up to 40% risk heterogeneity documented for mortality. The high proportion of missing SES data required imputation in a setting where data was not missing at random. Thus, we cannot be certain that excluding SES from mortality risk adjustment is correct. For the other outcome models, SES was missing at random.

Almost 20 years ago, Iezzoni maintained that answering the question on how to isolate quality-of-care differences requires expensive, time-consuming, logistically difficult, and methodologically complicated research and that only a handful of studies had addressed this question, most with equivocal results [34]. Risk adjustment for quality improvement spans all medical disciplines from alternative medicine to surgery and is performed by researchers, administrators, government agencies and health insurances. Yet we have not found a published solution on how to effectively isolate quality-of-care differences since Iezzoni’s comment other than approaches that entailed visiting the participating clinics and assessing the attributional validity of their risk adjustment model on site, such as the DAVROS group [13]. But besides having its own bias issues, this approach, requiring independent experts, is personnel and time intensive. We could not afford copying it for one outcome let alone seven. It is therefore not so surprising that most of the literature on risk adjustment relies on predictive validity alone and measures neither change in attributional validity nor attributional validity itself. It would no less be important to know if quality improvement collaboratives with larger populations and/or differences in case-mix risk show a larger effect of risk adjustment and more effectively isolate quality differences.

## Conclusions

To summarize, all three approaches to risk adjustment revealed comparable results with high predictive validity for mortality and several major morbidities. The indirect standardization approach was surprisingly similar to the other approaches.

In general, however, we conclude that risk adjustment has a limited effect when comparing the outcome of clinics with the same degree of specialization. We therefore propose that clinic outcomes be compared both in risk-adjusted and un-adjusted form. The predictive validity of the risk-adjustment should be quantified and reported. To correct for confounding, stratification into risk groups (such as gestational age or sex) could be used as an alternative to risk-adjustment. Rather than relying on the purely methodological approach of risk-adjustment alone, as done ubiquitously by organizations delivering risk-adjusted annual reports, our experience shows that building small groups of clinic representatives and combining their expertise with methodologically transparent presentation of epidemiological data is very helpful in isolating relevant quality improvement potential.

## Additional file

Additional file 1: Table S1. Description: Comparison between expected events when calculated by logistic regression using GA alone to approximate indirect standardization stratified by gestational age weeks for 2013–2014. (DOCX 17 kb)

## Abbreviations

AUC: Area under receiver operating characteristics curve
BPD: Bronchopulmonary dysplasia
BW z-score: Birthweight z-score
GA: Gestational age
GA2: GA squared
N: Sample size for analysis
NEC: Necrotizing enterocolitis
SES: Socio economic status
sIVH: Intra−/periventricular haemorrhage grade 3–4
SMR: Standardized mortality/morbidity ratio
sROP: Retinopathy of prematurity grade 3 and above
