# Supplementary material for: Comparison of three different methods for risk adjustment in neonatal medicine
Source: BMC Pediatr. 2017 Apr 17;17:106. doi: 10.1186/s12887-017-0861-5 (PMC5392992; doi:10.1186/s12887-017-0861-5)
Supplement: Additional file 1: Table S1. — Description: Comparison between expected events when calculated by logistic regression using GA alone to approximate indirect standardization stratified by gestational age weeks for 2013–2014. (DOCX 17 kb) [file 12887_2017_861_MOESM1_ESM.docx]

Table S1 *Comparison between expected events when calculated by logistic regression using GA alone to approximate indirect standardization stratified by gestational age weeks for 2013-2014.*

| Outcome | N | True  events | Logistic  regression | Indirect  standardization | Difference^[[1]](#endnote-1)^ |
| --- | --- | --- | --- | --- | --- |
| Overall mortality | 1572 | 220 | 244.77 | 251.47 | 0.43% |
| Inhosp. Mortality | 1465 | 113 | 131.31 | 133.69 | 0.16% |
| Late onset sepsis | 1465 | 119 | 140.29 | 137.28 | -0.21% |
| NEC^[[2]](#endnote-2)^ | 1465 | 35 | 35.49 | 35.21 | -0.02% |
| sIVH^[[3]](#endnote-3)^ | 1459 | 83 | 96.64 | 97.62 | 0.07% |
| BPD^[[4]](#endnote-4)^ | 1349 | 151 | 127.12 | 125.13 | -0.15% |
| sROP^[[5]](#endnote-5)^ | 1200 | 19 | 25.99 | 24.96 | -0.09% |

1. Difference (%) represents difference between the two approaches in relationship to population size (N). [↑](#endnote-ref-1)
2. NEC: Necrotizing enterocolitis. [↑](#endnote-ref-2)
3. sIVH: intra-/periventricular hemorrhage grade 3-4. [↑](#endnote-ref-3)
4. BPD: bronchopulmonary dysplasia. [↑](#endnote-ref-4)
5. sROP: retinopathy of prematurity grade 3 and above. [↑](#endnote-ref-5)
